# Medical Aristocracy

**Published:** 1888-08

**Authors:** 


MEDICAL ARISTOCRACY is the caption of an article of the correspondent of the *Detroit Tribune* in his account of the late meeting of the American Medical Association, the action of which he styles "High-handed Propositions," and states that the American Medical Association, with nearly fiteen hundred members present, of whom twenty-five are women, held a preliminary session in Music Hall to-day, at which welcoming oratory was supplied by Mayor Smith, on behalf of the city, and by Dr. C. D. Comegys, on behalf of the local medical fraternity. President Garnett replied and also submitted his annual address, which was distinguished for its demand for reform. His idea was to appoint a committee of five in each State to attend meetings of the legislature and secure legislation for the following objects:

To reduce the number of medical schools and graduates by more stringent charter and matriculation and graduation laws. Laws providing for stringent examinations of would be practitioners by a competent medical board before being granted a license to practice, the applicant's diploma not being taken for evidence of competency. That heavy penalties be provided for any physician found practicing without a license from this state board. That the faculties of the several medical schools within the limits of the United States be urgently requested to call a convention at some central point for the purpose of consultation and adopting some general and uniform system of medical education more comprehensive and rigid in its requirements and more in accord with the spirit of the age and the advanced progress of medical science, suggesting a four years' term of study, the requirements of a preliminary education including some knowledge of the classics. That any college or school which shall refuse to enter into such an arrangement as may be decided upon by said convention shall be excluded from all connection with the American Medical Association, and its alumni not recognized as members of the regular profession.

